# Supplementary material for: “A net for everyone”: fully personalized and unsupervised neural networks trained with longitudinal data from a single patient
Source: BMC Med Imaging. 2023 Oct 31;23:174. doi: 10.1186/s12880-023-01128-w (PMC10619304; doi:10.1186/s12880-023-01128-w)
Supplement: Supplementary file 1 — Supplementary Material 1 [file 12880_2023_1128_MOESM1_ESM.docx]

# Supplementary Materials


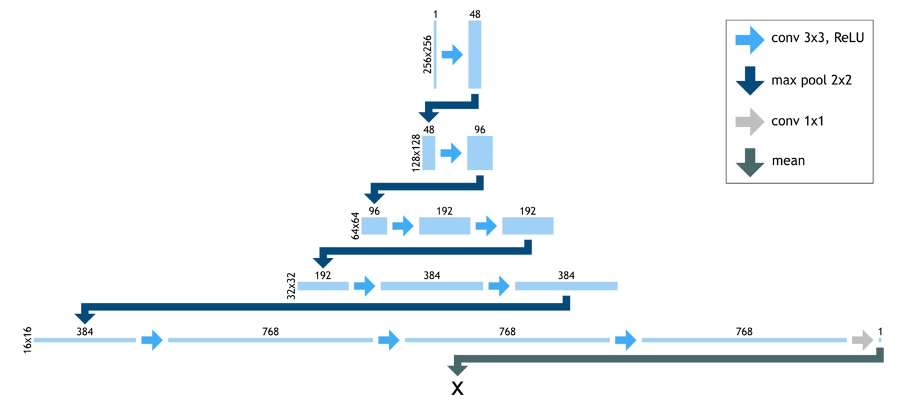


Figure S1: Architecture of the discriminator network. An encoder structure is used. The image size at each level is shown on the left of the blocks, the feature size is shown on top of the blocks. The number of 3x3 convolutions at each level increases further down the network. 2x2 max pool functions are used for downsizing. At the last level, the number of features are decreased to one with a 1x1 convolution. This last block is averaged to get the single value output of x.


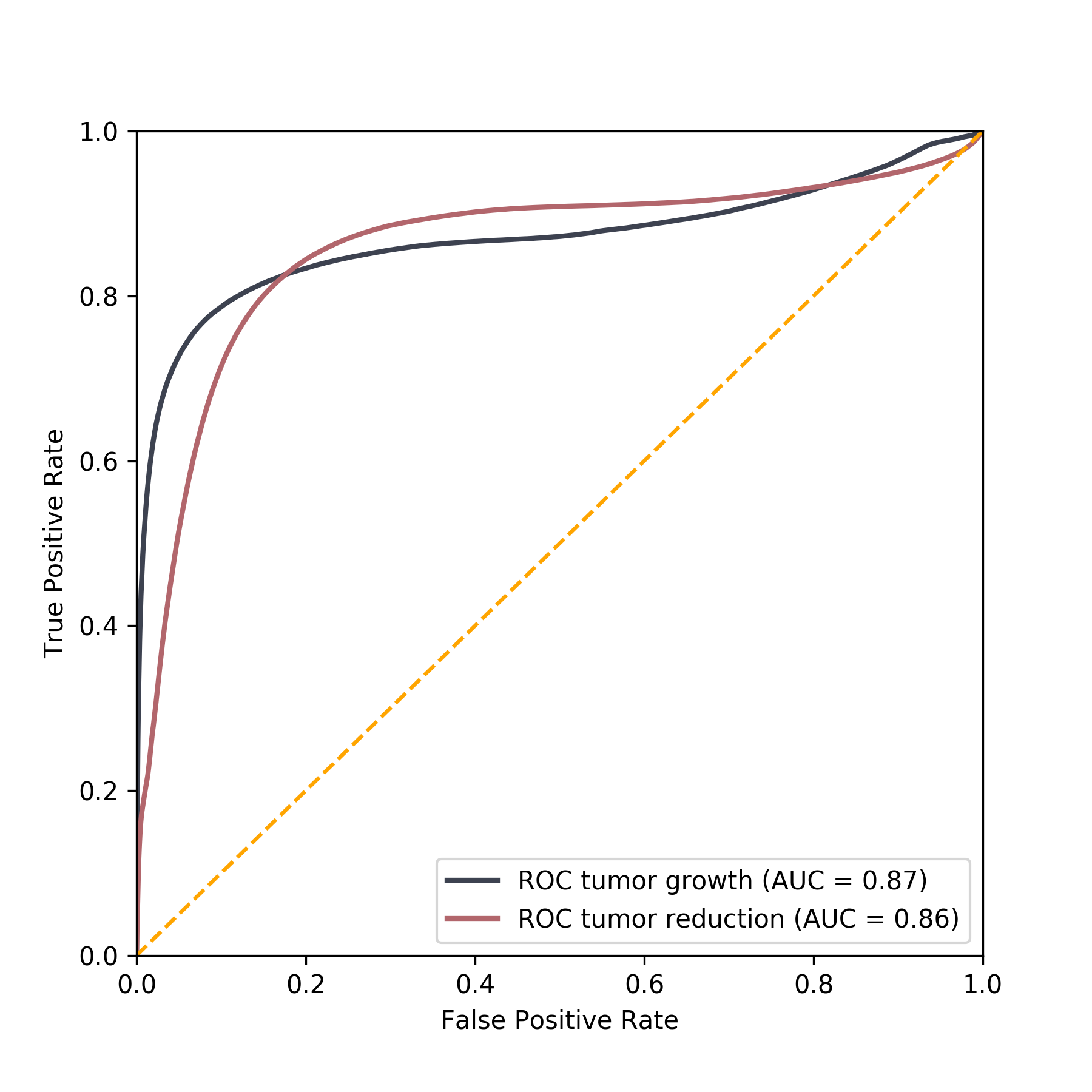


Figure S2: ROC analysis for prediction of tumor change compared against BraTS winning network nnUnet for both datasets combined.

| **RANO category** | **Public Dataset** | | **Private Dataset** | |
| --- | --- | --- | --- | --- |
|  | **Sensitivity** | **Specificity** | **Sensitivity** | **Specificity** |
| Response | 66.7% | 100.0% | 66.7% | 100.0% |
| Stable disease | 85.7% | 50.0% | 66.7% | 72.7% |
| Progression | 42.9% | 90.0% | 60.0% | 77.8% |
| **Total** | **64.7%** | **82.4%** | **64.3%** | **82.1%** |

Table S1: Sensitivity, Specificity for each dataset.
